# Effect of Delivery by Emergency or Elective Cesarean Section on Nitric Oxide Metabolites and Cortisol Amniotic Concentrations in at Term Normal Newborn Dogs: Preliminary Results

**DOI:** 10.3390/ani11030713

**Published:** 2021-03-05

**Authors:** Jasmine Fusi, Augusto Carluccio, Tanja Peric, Massimo Faustini, Alberto Prandi, Maria Cristina Veronesi

**Affiliations:** 1 Department of Veterinary Medicine, Università degli Studi di Milano, 26900 Lodi, Italy; jasmine.fusi@unimi.it (J.F.); massimo.faustini@unimi.it (M.F.); maria.veronesi@unimi.it (M.C.V.); 2 Faculty of Veterinary Medicine, University of Teramo, 64100 Teramo, Italy; acarluccio@unite.it; 3 Department of Agricultural, Food, Environmental and Animal Sciences, University of Udine, 33100 Udine, Italy; alberto.prandi@uniud.it

**Keywords:** newborn dog, elective Caesarean section, emergency Caesarean section, fetal fluid, nitric oxide metabolites, cortisol

## Abstract

**Simple Summary:**

The high perinatal mortality rates in dogs are partly attributable to stress at parturition, with the production of cortisol (C), and related to the type of delivery, that is elective or emergency cesarean sections (ELCS and EMCS). Nitric oxide metabolites (NOs) are also related to the type of parturition (ELCS or EMCS), because of the different emotional and physical stresses experienced by the bitch in these two scenarios. The study aimed to assess the concentrations of C and NOs in the amniotic fluid of puppies delivered by ELCS or EMCS. In the amniotic fluid of the 32 puppies delivered by ELCS, C, and NOs concentrations were significantly lower than those found in the amniotic fluid of the 22 puppies delivered by EMCS. Lower C concentrations were found at increasing newborn viability assessed by Apgar score. Higher amniotic NOs concentrations were associated to increasing mother’s parity, puppies’ birthweight, and time of labor within the EMCS group. Due to the possible concurrence of several compartments (maternal, maybe placental, and fetal) to the final amniotic fluid composition, the definition of the role played by the three compartments in the higher C and NOs concentrations found in amniotic fluids collected from puppies delivered by EMCS than ELCS needs further clarifications.

**Abstract:**

The neonatal response to stress was reported to be related to the type of delivery, that is elective or emergency cesarean sections (ELCS and EMCS, respectively). Nitric oxide (NO) is also reported to be related to uterine inertia, and high levels of NO metabolites (NOs) are associated with physical and emotional stress. The study aimed to assess the concentrations of cortisol (C) and NOs in the amniotic fluid of puppies delivered by ELCS or EMCS. In total, 32 puppies were delivered by ELCS and 22 by EMCS. ANCOVA showed an effect of the ELCS vs. EMCS on both amniotic NOs (*p* < 0.001) and C (*p* < 0.001) concentrations. Lower amniotic C concentrations were found at increasing Apgar score (*p* < 0.001). Higher amniotic NOs concentrations were associated to increasing mother’s parity (*p* < 0.001), puppies’ birthweight (*p* < 0.001), and time of labor within the EMCS group (*p* < 0.05). A positive correlation between birthweight and amniotic NOs concentrations was also found (*p* < 0.05) in the EMCS group. Due to the possible concurrence of several compartments (maternal, maybe placental, and fetal) to the final amniotic fluid composition, the definition of the role played by the three compartments in the higher C and NOs concentrations found in amniotic fluids collected from puppies delivered by EMCS than ELCS needs further clarification.

## 1. Introduction

The high perinatal mortality rate reported in the dog [1,2] is caused by a multiplicity of factors, among which the process of whelping represents a major issue. As litter-bearing, the dog is characterized by a relatively long stage 2 labor, generally occurring within 2 to 12 h, but that may last as long as 24 h [3]. However, the survival rate of newborns was negatively correlated to the length of the stage 2 labor. Recent studies, in fact, provided evidence that stage 2 labor around 5.5 h are associated to the highest newborn survival rates [4]. Moreover, among the numerous canine breeds belonging to the dog species, some are considered at risk for dystocia, often caused by uterine inertia, especially for those breeds characterized by large litter-size, as for instance large-sized dogs breeds [5,6]. Dystocia is a concern because of possible risk for puppies’ mortality, as a consequence of fetal hypoxia.

From a physiologic standpoint, parturition is a process finely orchestrated by both the mother and the fetus(es). In this complex process, many factors are involved to prepare both the fetuses to be expelled and to shift to an “independent” life, to prepare the mother to deliver fetuses and placentas, to lactation, and to provide the maternal cares to the offspring. Cortisol (C) is one of the most studied hormones involved in the final fetal maturation [7] and neonatal adaptation [8], but also in the process of parturition triggering [9]. Cortisol is also known as the endpoint of stress, as its synthesis could be related to the activation of the hypothalamus-pituitary-adrenal axis [10].

Another important factor that contributes to the maintenance of pregnancy and to the onset of labor is nitric oxide (NO) [11]. Up to now, three isoforms of the enzyme responsible for the synthesis of NO (NO synthase, NOS) were identified in human myometrium: neuronal (nNOS), endothelial (eNOS), inducible (iNOS) [11,12]. Neuronal NOS was reported to have a role in maintaining normal pregnancy. Indeed, the increase in its expression in fetal tissue, followed by a decrease during spontaneous delivery, suggests that the increased production of NO helps to maintain uterine quiescence [11]. Nitric oxide metabolites, nitrate (NO_3_^−^) and nitrite (NO_2_^−^), together expressed as NOs, can be measured to assess NO production [11,13]. In a study by [11], in humans the amniotic fluid and maternal blood concentrations of NOs were reported to be higher in in abnormal than normal late pregnancies. In addition, NOs were found to be higher in amniotic fluid than in maternal plasma, suggesting an active role of the fetus in pregnancy regulation [11]. Very little is reported in veterinary medicine about NO, even if iNOS was reported to be associated with physical and emotional stress in rats [14].

The authors are not aware of studies investigating the maternal production of NOs in the bitch during pregnancy and at normal or disturbed parturition. It is however reasonable to suppose that, also in the dog, NOs could be involved in the process of pregnancy maintenance by uterine quiescence. Bitches with dystocia related to uterine inertia could therefore produce high NOs. Moreover, the bitches with dystocia experience both physical and emotional stress before the emergency Caesarean section (EMCS), due to the delayed and/or disturbed delivery of the first pup, or after one or more puppies have been delivered. This could probably affect both C and NOs synthesis by the maternal compartment. On the other hand, during dystocia, the fetuses also experience conditions of stress and hypoxia, with high secretion of C and possible synthesis of NOs. However, bitches at high-risk for dystocia can be submitted to elective Caesarean section (ELCS), at the physiologic term of pregnancy, before the onset of labor. In this case they probably do not experience the same stress conditions, with possible different impact on the maternal (and maybe fetal) synthesis of C and NOs. On the other hand, the not yet started labor could be related to still high maternal NOs synthesis.

The recent increasing awareness in animal welfare and limitations of disturbing procedures, encouraged the use of matrices collectable without invasiveness. With this purpose, to investigate the close perinatal period in the dog, some studies [15,16,17,18,19] focused on the collection of amniotic fluid at term, demonstrating the safety of the procedure and the usefulness of that matrix for a multiplicity of analyses.

Therefore, the present study was aimed to add new information about the possible different impact of ELCS or EMCS on the concentrations of NOs and C in amniotic fluid collected in normal dogs delivered at term by ELCS or by EMCS. The possible effects played also by some factors (maternal breed, age and parity, time of labor within the EMCS group, litter-size, newborn gender, birthweight, and Apgar score) on amniotic NOs and C concentrations were assessed.

## 2. Materials and Methods

The study was performed in agreement with the animal welfare committee ethical guidelines: all the procedures were carried out according to the Italian legislation on animal care (Decreto Legge 116, 27/01/1992) and also to the European Guidelines on Animal Welfare (Directive 2010/63/EU). A written informed consent was obtained by owners, not only to submit pregnant dogs to ELCS or EMCS, but also for the collection of clinical records and amniotic fluids for research purposes.

### 2.1. Animals

The study was performed on 13 large breeds bitches of private breeders (eight Maremma dogs, two German shepherds, one Boxer, one Newfoundland, one Rottweiler), aged 3–9 years old, at their second or third parturition.

In 5 bitches the medical history reported previous experience of dystocia due to uterine inertia and requiring EMCS, with neonatal losses. Those bitches were therefore scheduled for ELCS. Other 8 bitches did not show troubles at previous parturitions, but were admitted to surgery for dystocia occurrence in the present parturition.

All bitches were healthy and monitored by clinical and ultrasonographic exams, from the time of the sole mating, along the whole pregnancy, until approaching parturition.

### 2.2. Elective and Emergency Caesarean Sections

The dates of ELCSs were scheduled on the basis of the cumulative evaluation of several factors, previously reported [19]: the value of progesterone plasma concentrations at mating; the ultrasonographic measurement of the inner chorionic cavity at 25–28 days after mating; the ultrasonographic measurement of the fetal biparietal diameter at 40–45 days after mating. Moreover, mothers and fetuses wellbeing in the last week of pregnancy was daily checked, coupled to the measurement of plasma progesterone concentrations. As previously reported [15], ELCSs were performed when plasma progesterone concentrations fell below 2 ng/mL.

All the EMCSs were performed at dystocia occurrence. Dystocia was diagnosed in the following conditions: green or black vaginal discharge, unsuccessfully strains for more than 30 min, interval of maximum 3 h between puppies’ expulsion, presence of fetal distress (heart rate < 180 bpm), narrow birth canal, fetal obstruction, uterine inertia [6,20,21,22].

The anesthetic protocol and surgical procedures [18,23] were the same in ELCS and EMCS. Both anesthesia and surgery were aimed to minimize the side effects on newborn’s viability. Briefly, after premedication with metoclopramide and cefazolin, and oxygen mask provision, induction was obtained by propofol infusion and anesthesia maintenance in isoflurane and oxygen. Lidocaine infiltration on the line of surgical incision was also performed. Only after the extraction of the last fetus, tramadol and oxytocin were administered to the bitch.

The time elapsing between anesthesia induction and the extraction of the last fetus was recorded in both ELCS and EMCS. In EMCS, also the time elapsing between the actual beginning of the expulsion phase (observation of first abdominal strengths) and presentation for surgery was recorded.

### 2.3. Newborn Puppies’ Evaluation and Amniotic Fluid Collection

At extraction of fetuses, neonatal care and amniotic fluids collection were assured by two different expert veterinarians. Amniotic fluids, collected according to previous studies [15,16,17,18,19], were immediately centrifuged at 1000× *g* for 10 min [18]. The supernatant was removed and stored at −20 °C until analysis for C and NOs. Analysis were always performed within 3 months from fluid collection [17,18,24].

Newborns cares and evaluation were performed by expert neonatologists. Viability was assessed by Apgar score in all puppies only once, up to 5 min after birth, and newborns classified as viable when Apgar score was ≥7 [25]. According to Apgar score, each puppy was submitted to a different degree of neonatal assistance and/or resuscitation according to [25]. Newborns were also evaluated for the absence of gross malformations or physical defects. Parameters such as litter-size, newborn gender, birthweight, maternal age, and parity were recorded. Newborn survival and health status of bitches and puppies at 7 days after parturition were also recorded.

### 2.4. Cortisol and NOs Analysis

The analysis of C in amniotic fluids was performed according to the procedure described by [17]. A Radio-Immuno Assay (RIA) technique for cortisol concentrations (Cortisol, [1,2,6,7-3H (N)] Perkin-Elmer Life Sciences, Boston, MA, USA) by Top-Count (Perkin-Elmer Life Sciences, Boston, MA, USA) was used. The intra-assay and inter-assay coefficients of variation were 4.8 and 8.7%, respectively. The sensitivity of the assay was 2.94 pg/well. The relationship between the amniotic fluid C and standard C curve determined through linear regression was linear—the correlation coefficient r was 0.99 and the model was given by the equation y = 0.9490x + 0.279.

Amniotic fluid samples were thawed on ice and were ultrafiltered through a 10 kDa molecular weight cut-off filter (Amicon^®^ Ultra 0.5 mL Centrifugal filters, Merck KGaA, Darmstadt, Germany). The filters were pre-rinsed with ultrapure water prior to ultrafiltration of samples. Ultrafiltration reduces background absorbance due to the presence of hemoglobin and improves color formation using the Griess Reagents (Merck KGaA). Nitrate (NO_3_^−)^ and nitrite (NO_2_^−^) were assayed using a commercial kit (Cayman Chemical, Ann Arbor, MI, USA) in 80 µL of samples of amniotic fluid. This assay kit measures total nitrate and nitrite concentration in two-step. The first step is the conversion of nitrate to nitrite utilizing nitrate reductase. The second step is the addition of the Griess Reagents which convert nitrite into a deep purple azo compound. Photometric measurement of the absorbance due to this azo chromophore accurately determines NO_2_^−^ concentration. In brief, 10 µL of enzyme cofactors and 10 µL of nitrate reductase were added to 80 µL of standard and sample in 96-well solid plate. After the incubation of 3 h at room temperature, 50 µL of sulfanilamide (Griess Reagent 1) followed by 50 µL of N-(1-naphthyl)-ethylenediamine (Griess Reagent 2) were added and the color was allowed to develop for 10 min at room temperature. Reagent blanks were prepared for each sample by adding 100 µL of assay buffer instead of the Griess reagents. The absorbance was determined at 540 nm using a plate reader (Ensight Multimode plate reader, Perkin-Elmer Life Sciences). For each sample, a correction was made for the absorbance of the corresponding reagent blank. A serially diluted nitrate standard curve was linear over the range 2.5 to 35 µL. The conversion of nitrate to nitrite was almost 100%. The inter- and intra-assay coefficients of variation were 8.3 and 3.8%, respectively.

### 2.5. Statistical Analysis

To assess the possible effect played by the type of delivery (ELCS or EMCS) and by the above mentioned maternal and neonatal factors on amniotic NOs and C concentrations, data were submitted to an ANCOVA test, considering the type of parturition (EMCS or ELCS), newborn gender (male or female), and breed (Maremma vs. other breeds) as fixed factors, and mother’s age and parity, litter-size, puppy birthweight, and Apgar score as covariates.

Aiming to detect the influence of variables in the ANCOVA model, a multiple linear regression model was applied, considering amniotic NOs or C concentrations as response variables, and type of delivery (EMCS and ELCS), breed, mother’s age and parity, litter-size, puppy birthweight, and Apgar score as explicative variables. Due to the fixed nature of the type of delivery (EMCS and ELCS), ELCS was chosen as reference group.

The one-way ANOVA was used to assess the possible influence of time of labor within the EMCS group on amniotic NOs concentrations.

Moreover, the Pearson correlation index r was calculated between amniotic NOs and C concentrations within EMCS and ELCS, and also between the puppies’ birthweight and amniotic NOs concentrations.

## 3. Results

### 3.1. Clinical Findings

In the ELCS group the interval of time between anesthesia induction and the last fetus extraction was 24 ± 9.62 min.

In the EMCS group, the found causes of dystocia were: uterine inertia (5 bitches), fetal obstruction (1 bitch), fetal death (1 bitch), and unsuccessfully strains for more than 30 min after the expulsion of the previous puppy (1 bitch). Among the 25 spontaneously puppies delivered before surgery, 4 (16%) were dead.

In the EMCS group, the time elapsing between the beginning of parturition and presentation for surgery ranged between 4 and 6 h. In this group of bitches, the number of fetuses remaining to be delivered ranged between 1 and 5. In the EMCS group the interval of time between anesthesia induction and last fetus extraction was 19.38 ± 3.74 min.

A total of 54 puppies were obtained: 22 (10 females and 12 males) delivered by EMCS; 32 puppies (16 females and 16 males) were delivered by ELCS.

All puppies were healthy, viable and with no malformations or physical defects. The control at 7 days after parturition showed that all the puppies were alive and healthy at 7 days of age, and that all mothers were healthy and with normal post-partum course.

In Table 1 the maternal and neonatal clinical findings related to the 5 ELCS and to the 8 EMCS, expressed as mean ± SD and (min-max), are reported.

### 3.2. Amniotic NOs and C Concentrations

The ANCOVA test showed an effect of the type of delivery (ELCS vs. EMCS) on both amniotic NOs (*p* < 0.001) and C (*p* < 0.001) concentrations, with higher NOs and C concentrations in amniotic fluids collected by puppies delivered by EMCS.

The descriptive data (mean ± SD, min-max) about amniotic NOs and C concentrations in the 32 puppies delivered by ELCS and in the 22 puppies delivered by EMCS are reported in Table 2.

The ANCOVA test showed also an effect of the covariate Apgar score (*p* < 0.001) only on amniotic C concentrations, but not on amniotic NOs concentrations, with lower C concentrations associated to increasing Apgar score.

The data, expressed as mean ± SD, about amniotic NOs and C concentrations distributed according to Apgar score, in the 32 puppies delivered by ELCS and in the 22 puppies delivered by EMCS, are reported in Table 3.

Moreover, the ANCOVA highlighted an effect of the covariate maternal parity (*p* < 0.001) on amniotic NOs, but not C, concentrations, with higher amniotic NOs concentrations associated to increasing maternal parity.

The data expressed as mean ± SD, about amniotic NOs and C concentrations distributed according to maternal parity in the 32 puppies delivered by ELCS and in the 22 puppies delivered by EMCS are reported in Table 4.

The one-way ANOVA showed a significant association (*p* < 0.05) between increasing time in labor before surgery in EMCS group and amniotic NOs concentrations. In fact, in bitches in which only 4 h lasted between labor beginning and surgery, the amniotic NOs concentrations were 43.2 ± 22.5 vs. 68.7 ± 39.7, and 169.8 ± 106.5 μmol/L when the time of labor was 5 and 6 h, respectively.

The multiple linear regression showed an association (*p* < 0.001) between amniotic NOs concentrations and puppies’ birthweight, with higher amniotic NOs concentrations associated to increasing puppies’ birthweight. The Pearson test highlighted a positive correlation between birthweight and amniotic NOs concentrations (r = 0.5; *p* < 0.05), but only within the EMCS group.

On the contrary, no significant effects played nor on C, neither on NOS amniotic concentrations, were found for other covariates, such as maternal age, breed, newborn gender and litter-size.

The Pearson correlation test did not show significant correlation between NOs and C amniotic concentrations, nor within the ELCS, neither in the EMCS group.

## 4. Discussion

In literature there is a lack of knowledge about the role of NOs changes occurring during normal or disturbed parturition, and scarce data about the impact of type of delivery on amniotic C concentrations. For these reasons, the present study aimed to assess possible differences in amniotic NOs and C concentrations in normal newborn dogs born by ELCS or EMCS.

Because both C and NOs could be synthesized by the maternal, placental, or fetal compartment, it could have been interesting to measure not only the amniotic fluid, but also the maternal blood, as reported in humans [11].

However, although all the bitches were vein cannulated for surgery, the collection of blood samples for research purpose was not allowed by some of the owners, preventing the use of maternal blood for the analysis of plasma C and NOs concentrations.

A first consideration should be addressed to the clinical differences between ELCSs and EMCSs. In the present study, in fact, ELCSs were performed when the plasma progesterone concentrations were already decreased below the threshold of 2 ng/mL, indicating the onset of the physiologic process of parturition, even if the stage 2 labor of parturition was not yet started. Therefore, although according to [26,27] the ELCS could be considered as “parturient” ELCS, in all the cases the surgery was performed before stage 2 labor beginning. This means that the massive changes related to that stage 2 labor were not yet started. The EMCSs, on the opposite, were performed when parturition was already started, as evidenced by previous expulsion of puppy(ies) or by the observation of unsuccessful abdominal strains for more than 30 min. Taken together, these conditions highlight that surgery was performed during the stage 2 labor.

However, according to the chosen clinical management, after a clinical examination evidencing a possible dystocia, no attempts of manual or pharmacological assistance were done, and a surgical approach was chosen with the aim to maximize newborn survival and avoid stressful conditions for the mothers [28]. This approach implies that the physiologic differences between the two types of parturition should be mainly addressed to the initiated or not initiated stage 2 labor. In this context, the results of the present study seem to suggest that the already started stage 2 labor entails higher concentrations of both NOs and C in the amniotic fluid collected during surgery.

Considering that canine amniotic composition is supposed to result from both the maternal and fetal compartment concurrence [16], the higher amniotic C concentrations in EMCSs could be related to the different maternal stress, or at least discomfort, and to the fetal distress, associated to EMCS.

However, the neonatal viability, assessed by Apgar score, showed high newborn viability in puppies delivered by both EMCS and by ELCS. This finding suggests that when a prompt Caesarean section is performed as an emergency, the fetal condition could be not endangered. Moreover, also the outcome at 7 days after delivery showed that all the newborns were alive and healthy, and bitches healthy and displaying normal post-partum. Therefore, the actual impact of higher amniotic C and NOs concentrations associated to ELCS, on newborn and maternal outcome, need further clarifications.

In the present study, the amniotic mean C concentrations found in the puppies delivered by ELCS was higher than the 3.7 ng/mL median C concentration reported by Bolis and coworkers [17]. Other than this, the amniotic C concentrations in puppies delivered by ELCS (5.85 ± 2.96 ng/mL) was slightly lower than the one reported by Melandri and coworkers in puppies delivered by ELCS (6.7 ± 3.3 ng/mL) [24]. On the other hand, the amniotic C concentrations in puppies delivered by EMCS (10.7 ± 4.15 ng/mL) was higher than those found by Groppetti and colleagues in (3.5 ± 1.4 ng/mL) [29].

The finding of a significant association between lower amniotic C concentrations and increased Apgar score seems to further suggest that, beside the Apgar score, also the measurement of amniotic C concentrations could represent an additional tool for newborn puppies’ evaluation, useful for better detecting those puppies needing a special surveillance early after birth, as previously reported [17]. However, as stated above, all the puppies were normal viable at birth and were alive and healthy at 7 days after parturition, so that the actual influence of lower amniotic C concentrations on newborn dogs’ viability should be better investigated, and possibly addressed to the changes occurring close around birth.

In humans being, higher amniotic NOs concentrations were reported in late abnormal than in normal pregnancies [11]. Higher NOs concentrations were found in the blood of women suffering of post parturient hemorrhage, supposed to be related to a possible uterine atony [30], as NO can signal smooth muscle relaxation and consequent uterine atony [30]. In addition, in women, higher blood NO concentrations were reported to be associated to the longer duration of labor and stress, measured as the time interval between the onset of labor, to the hospital admission [30]. Although it is not possible to compare the results obtained in the present study and data reported in humans [11], 6 out of 8 bitches undergoing EMCS were affected by uterine inertia, a condition that could have influenced the 2-fold higher amniotic NOs concentrations in EMCS than in ELCS group.

As compared with the amniotic NOs concentrations reported in humans, results from the present study showed higher mean amniotic NOs concentrations in both puppies delivered by ELCS (46.4 vs. 24.2 μmol/l in humans’ amniotic fluid collected at term, before the onset of labor, considered as elective Cesarean section) [11], and in puppies delivered by EMCS (93.9 vs. 39.2 μmol/l in human amniotic fluids collected at term in cases of abnormal pregnancies) [11].

In the present study, in addition to the type of surgery, some maternal and neonatal factors affected amniotic NOs concentrations. In fact, significantly higher amniotic NOs concentrations were found in 3rd than in 2nd parity bitches. Although in humans a possible different influence of parity on amniotic NOs concentrations was observed, in the present study it could be only speculated that this finding could reflect a different uterine condition (maybe less myometrial efficiency) of bitches at their 3rd parturition in comparison to 2nd parity bitches.

In the EMCS group, a significant association between the longer time of labor before surgery and amniotic NOs concentrations, was found. This finding highlights, once more, the importance of a prompt EMCS after dystocia detection.

The positive correlation between amniotic NOs concentrations and puppies’ birthweight is difficult to explain, but a role of the at term fetus as NO (co)synthesizer could be hypothesized.

Due to the possible concurrence of maternal (maybe placental) and fetal compartments to the final amniotic fluid composition, it remains uncertain, and this is a limitation of the study, the different role of these three compartments in the higher C and NOs concentrations found in amniotic fluids collected from puppies delivered by EMCS than ELCS.

## 5. Conclusions

In conclusion, the results from the present study showed that amniotic C and NOs concentrations are higher in puppies delivered by EMCS than ELCS, although all the puppies were normal at birth and showed normal outcome at 7 days after delivery. Lower amniotic C concentrations were associated to higher Apgar score, although always within the range of normal newborn puppy’s viability. Interestingly, higher amniotic NOs were found in 3rd than 2nd parity bitches and was related to increased puppies’ birthweight. Due to the possible concurrence of several compartments (maternal, maybe placental, and fetal) to the final amniotic fluid composition, the definition of the role played by the three compartments in the higher C and NOs concentrations found in amniotic fluids collected from puppies delivered by EMCS than ELCS, needs further clarification.

## Figures and Tables

**Table 1:** Data expressed as mean ± SD and (min-max) about the maternal and neonatal clinical findings in elective Caesarean section (ELCS) and emergency Caesarean section (EMCS).

Type of Delivery	Mothers Age (ys)	Mothers Parity (*n*)	Total Newborns (*n*)	Litter-size (*n*)	Males/Females (*n*)	Birthweight (g)	Apgar Score
**ELCS**	5.3 ± 1.3 (3–7)	2.7 ± 0.5 (2–3)	32	7.2 ± 2.1 (4–10)	16/16	622 ± 190.2 (260–950)	8.8 ± 1.1 (7–10)
**EMCS**	4.7 ± 1.7 (3–9)	2.1 ± 0.4 (2–3)	22	8.2 ± 3.1 (3–14)	12/10	551 ± 139.3 (300–760)	8.8 ± 1.2 (7–10)

**Table 2:** Descriptive data expressed as mean ± SD and (min-max) about NOs and C amniotic concentrations in the 32 puppies delivered by elective Caesarean section (ELCS) and in the 22 puppies delivered by emergency Caesarean section (EMCS).

Type of Delivery	NOs (μmol/L)	C (ng/mL)
ELCS (*n* = 32)	46.4 ± 31.3 ^a^ (1.86–125)	5.9 ± 3.0 ^a^ (1.40–11.8)
EMCS (*n* = 22)	94 ± 84.6 ^b^ (8.31–320)	10.7 ± 4.2 ^b^ (4.52–19.4)

^a,b^ denotes within column statistical significance (*p* < 0.001).

**Table 3:** Data (mean ± SD) about NOs and C amniotic concentrations distributed according to Apgar score in the 32 puppies delivered by elective Caesarean section (ELCS) and in the 22 puppies delivered by emergency Caesarean section (EMCS).

Amniotic Parameter	Apgar Score							
	10	10	9	9	8	8	7	7
	ELCS (*n*= 17)	EMCS (*n*= 8)	ELCS (*n*= 10)	EMCS (*n*= 4)	ELCS (*n* = 0)	EMCS (*n*= 6)	ELCS (*n*= 5)	EMCS (*n*= 4)
C (ng/mL)	2.6 ± 1.0	8.2 ± 4.2	3.2 ± 1.7	8.5 ± 3.4	-	8.7 ± 2.8	8.3 ± 2.3	14.5 ± 2.6
NOs (μmol/L)	71.1 ± 42.6	33.9 ± 18.7	53 ± 13.7	36.7 ± 29.2	-	27.4 ± 0.7	84.8 ± 29.6	188.8 ± 100.2

**Table 4:** Data (mean ± SD) about NOs and C amniotic concentrations distributed according to maternal parity in the 32 puppies delivered by elective Caesarean section (ELCS) and in the 22 puppies delivered by emergency Caesarean section (EMCS).

Amniotic Parameter	Parity			
	2nd	2nd	3rd	3rd
	ELCS (*n* = 10)	EMCS (*n* = 19)	ELCS (*n* = 22)	EMCS (*n* = 3)
C (ng/mL)	7 ± 2.3	11 ± 4.3	5.4 ± 3.1	8.4 ± 2.1
NOs (μmol/L)	26.6 ± 19.6	73.3 ± 52.9	53.7 ± 32	217.6 ± 145.1

## Data Availability

Data are available on request to the authors.
